# Assessment of different classification systems for predicting the risk of superior laryngeal nerve injury during thyroid surgery: a prospective cohort study

**DOI:** 10.3389/fendo.2023.1301838

**Published:** 2023-11-22

**Authors:** Changlin Li, Jiao Zhang, Gianlorenzo Dionigi, Hui Sun

**Affiliations:** ^1^ Division of Thyroid Surgery, China-Japan Union Hospital of Jilin University, Jilin Provincial Key Laboratory of Surgical Translational Medicine, Jilin Provincial Engineering Laboratory of Thyroid Disease Prevention and Control, Changchun, Jilin, China; ^2^ Division of Surgery, Istituto Auxologico Italiano Istituto di Ricovero e Cura a Carattere Scientifco (IRCCS), Milan, Italy; ^3^ Department of Medical Biotechnology and Translational Medicine, University of Milan, Milan, Italy

**Keywords:** Cernea classification, Kierner classification, Friedman classification, the external branch of the superior laryngeal nerve, intraoperative nerve monitoring

## Abstract

**Background:**

A multitude of anatomical variations have been noted in the external branch of the superior laryngeal nerve (EBSLN). In this study, intraoperative neuromonitoring (IONM) was used to assess the potential value of the different classical EBSLN classifications for predicting the risk of EBSLN injury.

**Methods:**

In total, 136 patients with thyroid nodules were included in this prospective cohort study, covering 242 nerves at risk (NAR). The EBSLN was identified by observing the cricothyroid muscle twitch and/or typical electromyography (EMG) biphasic waveform. The EBSLNs were classified by Cernea classification, Kierner classification, and Friedman classification, respectively. The EMG parameters and outcomes of vocal acoustic assessment were recorded.

**Results:**

The distribution of Cernea, Kiernea, and Friedman subtypes were, respectively, Cernea 1 (40.9%), Cernea 2A (45.5%), Cernea 2B (10.7%), Kierner 1 (40.9%), Kierner 2 (45.5%), Kierner 3 (10.7%), Kierner 4 (2.9%) and Friedman 1 (15.7%), Friedman 2 (33.9%), Friedman 3 (50.4%). The amplitudes of EBSLN decreased significantly after superior thyroid pole operation, respectively, in Cernea 2A (193.7 vs. 226.6μV, *P*=0.019), Cernea 2B (185.8 vs. 221.3μV, *P*=0.039), Kierner 2 (193.7vs. 226.6μV, *P*=0.019), Kierner 3 (185.8 vs. 221.3μV, *P*=0.039), Kierner 4 (126.8vs. 226.0μV, *P*=0.015) and Friedman type 2 (184.8 vs. 221.6μV, *P*=0.030). There were significant differences in F_max_ and F_range_ for Cernea 2A (*P=*0.001, *P=*0.001), 2B (*P=*0.001, *P=*0.038), Kierner 2 (*P=*0.001), Kierner 3 (*P=*0.001, *P=*0.038), and Friedman 2 (*P=*0.004, *P=*0.014). In the predictive efficacy of EBSLN injury, the Friedman classification showed higher accuracy (69.8% vs. 44.3% vs. 45.0%), sensitivity (19.5% vs. 11.0% vs. 14.0%), and specificity (95.6% vs. 89.9% vs. 89.9%) than the Cernea and Kierner classifications. However, the false negative rate of Friedman classification was significantly higher than other subtypes (19.5% vs. 11.0% vs. 14.0%).

**Conclusion:**

Cernea 2A and 2B; Kierner 2, 3, and 4; and Friedman 2 were defined as the high-risk subtypes of EBSLN. The risk prediction ability of the Friedman classification was found to be superior compared to other classifications.

## Introduction

Intraoperative neural monitoring (IONM) is applied to explore the effects of vocal cord (VC) movement, voice quality, and swallowing during thyroid surgery. Quality of voice, breathing, and swallowing represent the final asset of the vagal nerve (VN), recurrent laryngeal nerve (RLN), and external branch of the superior laryngeal nerve (EBSLN) system. The intact RLN function is the prerequisite of intact VC function. However, intraoperative assessment of VN and RLN function may not be identical to VC movement and quality of voice. For this reason, there is an increasing consideration of the potential role of EBSLN identification, monitoring, functional preservation, and evaluation (1, 2).

EBSLN damage mainly leads to cricothyroid muscle (CTM) paralysis, and its clinical symptoms and signs are mild, which can be easily misdiagnosed as laryngeal edema, vocal cord edema, and pharyngolaryngitis. In unilateral EBSLN damage, the vocal cord tension is weakened. When vocalizing, there may be a decrease in pitch, narrowing of the range, low voice, shortening of phonation time, inability to speak loudly, and other phonological changes. When bilateral EBSLN is damaged, the change in tone and sound quality is more obvious, and the symptoms of reduced pitch and monotonous tone can occur. EBSLN injury is more likely to be overlooked when combined with RLN damage (1–3). In fact, unlike RLN injuries, the symptoms of EBSLN injuries are mostly manifested during applications, such as singers and announcers. The first detection of symptoms of EBSLN injuries was seen in a soprano singer (1–3). Because these voice changes are peculiar and variable, the diagnosis of EBSLN dysfunction is difficult to confirm based solely on clinical or endoscopic findings (3, 4). The EBSLN is anatomically categorized on the basis of its association with surrounding adjacent organs, including the superior thyroid vasculature (STV), hypopharyngeal constrictor, CTM, and thyroid cartilage. According to EBSLN closeness, different classifications have been proposed (**Table 1**) (5–7). The Cernea, Kierner, and Friedman schemes have been widely applied in clinical and research practice with the intention of identifying a risk condition.

**Table 1:** The classifications of EBSLN applied in the study.

Classification	Classification criteria			
Cernea (5) (Cadaver)	Type 1(60%):crossing STV more than 1 cm above the STP	Type 2A(17%):crossing STV less than 1 cm above the STP	Type 2B(20%):crossing STV below the STP	unidentified(3%)
Kierner (6) (Cadaver)	Type 1(42%):crossing STV more than 1 cm above the STP	Type 2(30%): crossing STV less than 1 cm above the STP	Type 3(14%):crossing STV below the STP	Type 4(14%);running dorsally to the STP
Friedman (7, 8) (Living and cadaver)	Type 1 (20%):running superficially to the ICM	Type 2(67%):partly running the superficial of ICM	Type 3(13%):running deep to the ICM	

STV, superior thyroid vessels; ICM, inferior constrictor muscle; STP, superior thyroid pole.

In this prospective clinical study, IONM was used to evaluate the value of the different EBSLN classifications for predicting EMG alterations and the risk of EBSLN injury.

## Materials and methods

### Patients

The protocol was approved by the Institutional Review Board of the China-Japan Union Hospital of Jilin University, Division of Thyroid Surgery, Changchun, China. Patients signed an informed consent form before surgery. The technical details of the protocol were explained to them because it is difficult to understand. Those with the presence of preoperative vocal cord fixation/weakening, history of neck surgery, preoperative presence of tumor invasion of nerves, refusal of a neuromonitoring catheter, and preoperative presence of altered tone were excluded from enrollment.

### IONM standards

IONM was offered in the intermitted mode of application (1, 9, 10). Endotracheal tube-based surface electrodes were applied (Trivantage EMG tube, Medtronic, Jacksonville, Florida, USA) connected to both audio and visual IONM systems (NIM-Response 3.0, Medtronic, Jacksonville, Florida, USA).

EBSLNs were stimulated using a single-use, incrementing prass stimulating probe, monopolar, standard flexible tip (product n.8225490, Medtronic, Jacksonville, Florida, USA), 100ms impulse duration, and 4Hz frequency. Since the maximum amplitude of the EBSLN is usually low, the EMG of the EBSLN is monitored by lowering the event threshold to 50μV. Peak-to-peak amplitudes of evoked EMG activities were read directly on the monitor screen and stored.

EMG amplitude profile may vary during intraoperative nerve monitoring because of variations in several variables unrelated to nerve status. For this reason, the following issues were continuously verified and standardized: (a) the type of induction or maintenance of anesthesia was the same for each patient; (b) endotracheal tube position was continuously verified with video laryngoscopy; (c) efforts to choose an EMG internal diameter (ID) tube with ideal contact with the VC; (d) no manipulation of the trachea and surrounding tissues during stimulation and monitoring; (e) efforts to maintain precise stimulation probe-nerve contact and invariable site of incitement; (f) dry surgical field and nerve ensheathed by fascia); and (g) no electrical cautery was used with bleeding vessels around the nerve.

### IONM technique

In the current study, the EBSLN was identified and monitored using the following scheme of Chinese guidelines of intraoperative neural monitoring during thyroid and parathyroid surgery (2023 edition) (1, 9, 10):

### Definitions

#### True positive EBSLN signal

CTM twitch and EMG response were references for EBSLN identification and STV dissection. This response serves as a true positive stimulation.

#### True negative

Stimulation of the upper pedicle that can be divided without cricothyroid twitch and EMG response. This response serves as a true negative stimulation. EBSLN was named differently according to different anatomical markers (**Table 1**). Under the guidance of IONM, dissection and functional identification were performed on potentially damaged nerves at risk (NAR). S1 was defined as the EBSLN stimulation at initial identification. S2 was the EBSLN stimulation after Superior thyroid artery (STA) ligation.

#### Loss of signal

LOS of the EBSLN was defined as an absence of CTM twitch combined with the previously obtained EMG signal and biphasic waveform.

### EBSLN outcomes measured

Parameters recorded for each NAR were: EBSLN (S1, S2), RLN (R1, R2), and VN (V1, V2) amplitudes and latency determinations; pre- and post-operative stroboscopy evaluation (L1, L2) (at 24 hours before surgery and on the first postoperative day); Pre-, intra- and postoperative needle EMG of the CTM were not performed.

### Vocal folds mucosal wave

In contrast to recurrent laryngeal nerve injury, when the EBSLN is injured, the overall movement of the vocal cords tends to remain unchanged. However, it should be noted that the vibrational characteristics of the vocal fold mucous membrane do undergo alterations, resulting in changes in timbre and vocal range. The unaffected cricothyroid muscle tension on the healthy side causes the anterior joint to shift towards the affected side, leading to an asymmetric mucosal wave. This change in wave propagation and mucosal wave pattern primarily manifests in the affected folds. In order to assess this phenomenon, we utilized the Japanese PENTAX VNL-1070STK 3.3mm electronic stroboscopic video laryngoscope system to evaluate specific vocal cord mucosal wave parameters. These parameters include maximum vibration frequency (F_max_), minimum frequency (F_min_), frequency range (F_range_), and maximum frequency duration (F_duration_). Acoustic evaluation of the vocal cords was conducted both pre-surgery (within 24 hours before the procedure) and post-surgery (on the first day following the operation).

### Statistical analysis

All patient data were collected prospectively in a Microsoft Office EXCEL spreadsheet. All data are expressed as mean and standard deviation (Mean ± SD) unless otherwise stated. Statistical analysis was performed using SPSS, 20.0 for Windows (SPSS Inc, Chicago-Ill, USA). The chi-square test was used for dichotomous or hierarchical data. A t-test was used for normally distributed measures and p < 0.05 was the level of significance.

## Results

### Patients

From July 2022 to August 2022, 26 patients were excluded because of missing data (16), loss to follow-up (8), or a refusal to participate (2).

**Figure 1:**
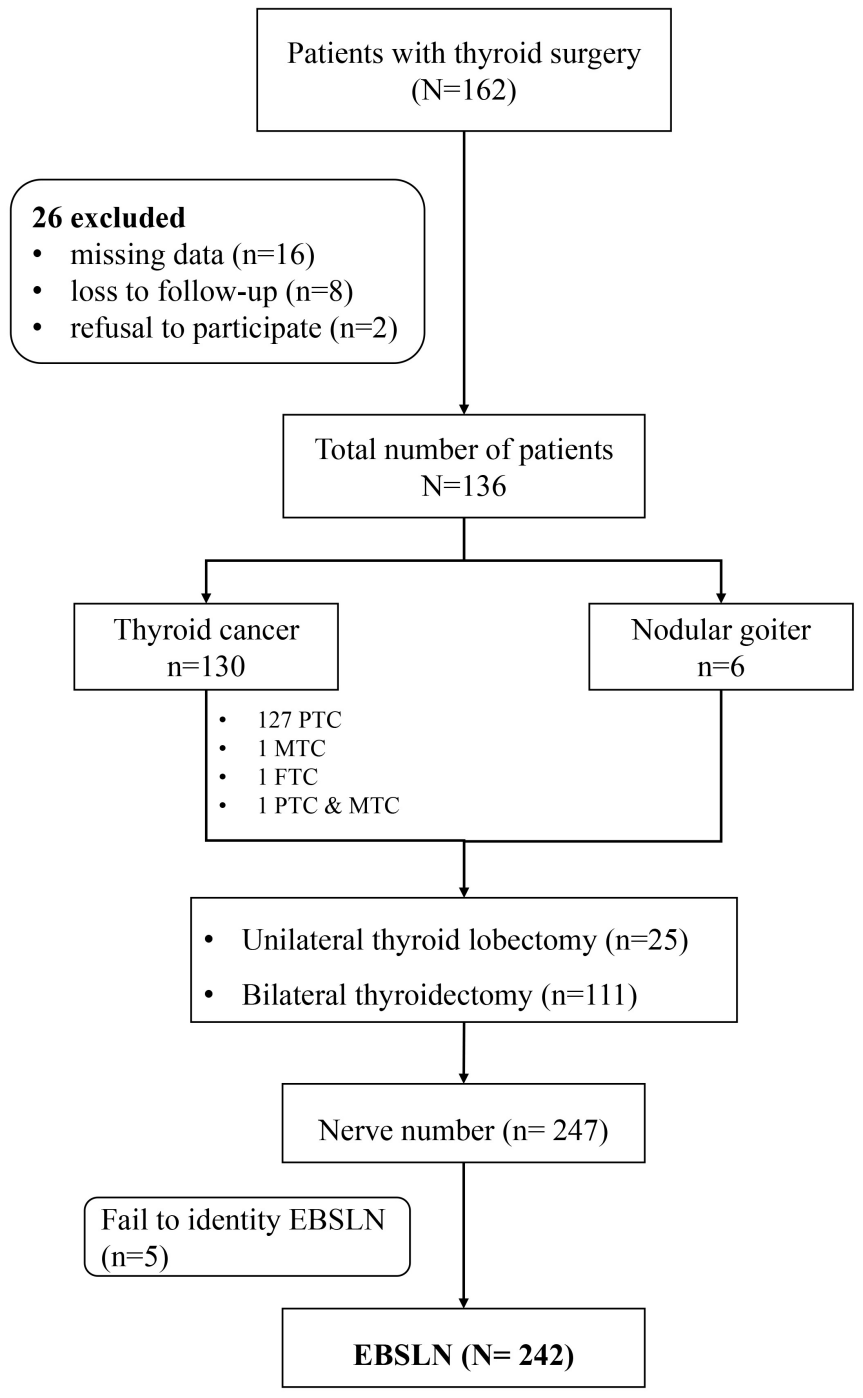
Flow chart of this study. EBSLN, the external branch of the superior laryngeal nerve.

In total, 136 patients with thyroid benign/malignant nodules were included in this prospective cohort study. There were 31 (22.2%) men and 105 (77.8%) women, their ages ranged from 21 to 66 years, with a mean age of 43.4 years. In all, there were 25 cases in which unilateral thyroid lobectomy was performed, and 111 cases in which bilateral thyroidectomy was performed, covering 242 NAR. Of these tumors, 127 (93.4%) were papillary thyroid cancer (PTC), 1 (0.7%) was a follicular tumor, 1 was (0.7%) medullary, 1 (0.7%) was coexisting PTC and MTC, and 6 (4.4%) were nodular goiter. Five NARs were not identified and 242 EBSLNs were analyzed (**Figure 1**).

### EBSLN monitoring

In total, 221 (91.3%) EBSLNs were identified by simultaneous twitch combined with EMG response and 21 (8.7%) EBSLNs were found by CTM twitch without clear EMG response. The mean initial amplitude (S1) of response for the EBSLNs was 224.5 ± 162.0 (62-932) μV.

The mean final amplitude (S2) was 193.4 ± 128.1 (53-754) μV. Two S2 determinations resulted in CTM twitch and EMG signal loss intraoperatively. Their S1 determinations were, respectively, 131 and 91 μV.

### Distribution of subtypes

The distribution of Cernea subtypes were 99(40.9%), 110 (45.5%), 26(10.7%), respectively for type 1, type 2A, and 2B; Friedman were 138(15.7%), 82(33.9%), and 122(50.4%) for type 1, type 2, and type 3; and Kierner were 99(40.9%), 110(45.5%), 26 (10.8%), and 7(2.9%) for type 1, type 2, type 3, and type 4 (**Table 2**).

**Table 2:** The distribution of EBSLN subtypes.

Cernea classification		Kierner classification		Friedman classification	
n (n/N %)		n (n/N %)		n (n/N %)	
*Type 1*	99 (40.9%)	*Type 1*	99 (40.9%)	*Type 1*	38 (15.7%)
*Type 2A*	110 (45.5%)	*Type 2*	110 (45.5%)	*Type 2*	82 (33.9%)
*Type 2B*	26 (10.7%)	*Type 3*	26 (10.7%)	*Type 3*	122 (50.4%)
*Unidentified (running dorsally to the STP)*	7 (2.9%)	*Type 4*	7 (2.9%)		

STP, superior thyroid pole.

### Amplitudes profiles

The amplitude values changed significantly from S1 to S2 for Cernea 2A and 2B (193.7 vs. 226.6μV, *P=*0.019; 185.8 vs. 221.3μV, *P=*0.039); Kierner 2, 3, and 4 (193.7 vs. 226.6μV, *P=*0.019; 185.8 vs. 221.3μV, *P=*0.039; 126.8 vs. 226.0μV, *P=*0.015); and Friedman 2 (184.8 vs.221.6μV, *P=*0.030) (**Table 3**). S1 and S2 amplitudes were similar for Cernea 1 (*P=*0.070), Kierner 1 (*P=*0.070), and Friedman 2 and 3 (*P=*0.078, *P=*0.072). The number of EBSLN with a decrease of amplitude exceeding 25% is significantly higher in Cernea 2B than in Cernea 1 and Cernea 2A (53.8% vs. 19.2% vs. 19.1%, P<0.001). In the Friedman classification, the number of EBSLN with a decrease exceeding 25% in Friedman 2 is significantly higher than in other subtypes (34.1% vs. 18.4% vs. 11.5%, *P*=0.035). Kierner 4 is revealed as the subtype with the highest proportion of decrease (71.4% vs. 53.8% vs. 19.2% vs. 19.1%, P<0.001). Similar trends are observed in subgroups with an amplitude decrease exceeding 50% (**Table 3**). 

**Table 3:** Longitudinal comparison of the amplitude profiles.

Classification	S1 (μV) Mean ± SD	S2 (μV) Mean ± SD	S2 25% decrease (N, %)	S2 50% decrease (N, %)	No CTM and LOS (N, %)
Cernea					
Type 1	226.4±164.9	200.3±135.1^NS^	19 (19.2%)	10 (10.1%)	0
Type 2A	226.6±165.6	193.7±123.5^*^	21 (19.1%)	12 (10.9%)	0
Type 2B	221.3±128.7	185.8±94.8^*^	14 (53.8%)^**^	3 (11.5%)^NS^	2 (7.7%)
Kierner					
Type 1	226.4±164.9	200.3±135.1 ^NS^	19 (19.2%)	10 (10.1%)	0
Type 2	226.6±165.6	193.7±123.5^*^	21 (19.1%)	12 (10.9%)	0
Type 3	221.3±128.7	185.8±94.8^*^	14 (53.8%)	3 (11.5%)	2 (7.7%)
Type 4	226.0±54.3	126.8±45.7^*^	5 (71.4%)^**^	5 (71.4%)^*^	0
Friedman					
Type 1	247.7±242.4	214.3±197.2 ^NS^	7 (18.4%)	0(0%)	0
Type 2	221.6±145.2	184.8±128.1^*^	28 (34.1%)	16 (19.5%)	2 (2.4%)
Type 3	217.1±166.6	192.9±123.4 ^NS^	14 (11.5%)^*^	7 (5.7%)^*^	0

Not indicated indicates no correlation.

^NS^ no significance.

^*^indicates significant correlation at level 0.05.

^**^ Significant correlation at 0.01 level.

Two S2 determinations resulted in CTM twitch and EMG signal loss intraoperatively, each of which belongs to high-risk subtypes of EBSLN (Cernea 2B and Friedman 2).

### Stroboscopy

Two nerves were confirmed EBSLN injuries by stroboscopy. The vibration of bilateral vocal cords was asymmetrical, irregular, and non-periodic, and the injured side was slightly bent, the length was shortened, and the tension was reduced.

### Vocal acoustic assessment

There were significant differences in F_max_ and F_ange_ for the Cernea 2A (*P=*0.001, *P=*0.001), Cernea 2B (*P=*0.001, *P=*0.038), Kierner 2 (*P=*0.001, *P=*0.001), Kierner 3 (*P=*0.001, *P=*0.038), Friedman 1 (*P=*0.015, *P=*0.030), and Friedman 2 (*P=*0.004, *P=*0.014) schemes (**Table 4**). There were no significant differences in F_max_, F_range_, and F_duration_ for Kierner subtype 4.

**Table 4:** Longitudinal comparison of the vocal acoustic assessment.

Classification	Pre-operative Mean ± SD			Post-operative Mean ± SD		
	F_max (HZ)_	F_range (HZ)_	F_duration (s)_	F_max (HZ)_	F_range (HZ)_	F_duration (s)_
Cernea						
Type 1	294.8 ± 39.0	119.6 ± 39.9	10.3 ± 6.4	275.8 ± 50.1^*^	119.8 ± 48.8	10.8 ± 5.5
Type 2A	294.4 ± 61.4	127.4 ± 47.8	9.2 ± 6.9	224.5 ± 42.1^***^	69.4 ± 26.5^***^	7.9 ± 4.4
Type 2B	325.2 ± 43.9	144 ± 39.1	6.3 ± 3.6	244.8 ± 22.1^**^	85.3 ± 30.8^*^	9.4 ± 2.2^**^
Kierner						
Type 1	294.8 ± 39.0	119.6 ± 39.9	10.3 ± 6.4	275.8 ± 50.1^*^	119.8 ± 48.8	10.8 ± 5.5
Type 2	294.4 ± 61.4	127.4 ± 47.8	9.2 ± 6.9	224.5 ± 42.1^***^	69.4 ± 26.5^***^	7.9 ± 4.4
Type 3	325.2 ± 43.9	144 ± 39.1	6.3 ± 3.6	244.8 ± 22.1^**^	85.3 ± 30.8^*^	9.4 ± 2.2^**^
Type 4	303 ± 60.8	93 ± 18.4	9.8 ± 5.6	248.5 ± 46.0	58 ± 9.9	11.1 ± 2.9
Friedman						
Type 1	320 ± 27.1	110.4 ± 45.8	10.0 ± 9.8	234.4 ± 55.5^*^	61.4 ± 33.1^*^	8.8 ± 6.9
Type 2	294.5 ± 63.3	125.6 ± 50.7	11.6 ± 6.5	242.3 ± 46.4^**^	88.1 ± 39.4^*^	10.3 ± 4.4
Type 3	300.3 ± 41.1	128.6 ± 30.8	6.8 ± 4.9	266.8 ± 48.2^**^	100.9 ± 42.8	8.0 ± 3.9

Not indicated indicates no correlation.

^*^indicates significant correlation at level 0.05.

^**^ Significant correlation at 0.01 level.

^***^ Significant correlation at 0.001 level.

F_max_, the maximum frequency; F_range_, range of frequency; F_duration_, maximum frequency duration.

### Horizontal comparison

The predictive efficacy of three types of classification for EBSLN injury was compared. There was no significant difference in the predictive ability of the Cernea and Kierner classifications for the risk of EBSLN injury, including accuracy (44.3% vs. 45.0%), sensitivity (11.0% vs. 14.0%), and specificity (89.9% vs. 89.9%) (**Table 5**). However, there was a significant difference in predicting the risk of EBSLN injury between the Friedman classification and the Cernea and/or Kierner classifications. The Friedman classification showed higher diagnostic accuracy (69.8% vs. 44.3% vs. 45.0%), sensitivity (19.5% vs. 11.0% vs. 14.0%), and specificity (95.6% vs. 89.9% vs. 89.9%). Moreover, the positive predictive value (69.6% vs. 60.0% vs. 66.7%) and negative predictive value (69.9% vs. 42.4% vs. 42.0%) of the Friedman classification were superior to other classifications (**Table 5**). However, the false negative rate of Friedman classification was significantly higher than other subtypes (19.5% vs. 11.0% vs. 14.0%).

**Table 5:** Prediction effectiveness of EBSLN risk.

	EBSLN Classification		
	Cernea	Kierner	Friedman
Accuracy (%)	44.3%	45.0%	69.8%^*^
Sensitivity (%)	11.0%	14.0%	19.5%^*^
Specificity (%)	89.9%	89.9%	95.6%^*^
FPR (%)	10.1%	10.1%	4.4%^*^
FNR (%)	11.0%	14.0%	19.5%^*^
PPV (%)	60.0%	66.7%	69.6%^*^
NPV (%)	42.4%	42.0%	69.9%^*^

FPR, false positive rate; FNR, false negative rate; PPV, positive predictive value; NPV, negative predictive value.

## Discussion

IONM with its EMG values obtained during surgery is similar to a new language that has to be learned: i.e., changes in amplitude, latency and waveforms, and postoperative assessments. Monitoring does not differ from other biological systems (1–3). **Table 1** describes the distribution of the Cernea, Kierner, and Friedman subtypes. In the Cernea classification reported in a cadaver study in 1992, type 1 (60%) was the most common subtype. The prevalent subtype in the Kierner classification was type 1 (42%) reported in 1998 in cadavers, and type 2 (67%) was the most frequent in the Friedman classification (5–7). In our clinical study, Cernea 2A (45.5%), Kierner 2 (45.5%), and Friedman 3 (50.4%) were more prevalent than other subtypes. The reason for these differences may be related to race, type of dissection, surgeon technique, cadaver use, underlying thyroid pathology, and the use of IONM.

Historically, the high-risk categories of EBSLN have been classified through clinical experience. Surgeons with their clinical experience thought that the EBSLN crossing the STV < 1 cm above the upper edge of the thyroid superior pole (Cernea 2A and 2B), or the nerve running in the pharyngeal muscle surface (Friedman 1), were at higher risk than other nerves.

Subsequently, surgeons defined the high-risk subtypes according to subjective indicators, such as the VHI-10 score and VII-5 score (11, 12). As we investigated in a previous article, the value of Cernea subtypes was assumed by EMG parameters for risk stratification (13). In the current study, we evaluated the risk of each EBSLN pattern by means of two objective indicators, both EMG parameters and vocal acoustic assessment.

By correlating the S1 amplitude with the S2 amplitude of different subtypes, amplitudes changed significantly from S1 to S2, respectively, in Cernea type 2A and 2B; Kierner type 2, type 3, and type 4; and Friedman type 2 (**Table 3**), which indicated that the above subtypes have a greater risk of EBSLN injury and were defined as the high-risk subtypes of EBSLNs. By examining the pre- and postoperative outcomes of vocal acoustic assessment, including F_max_, F_range_, and F_duration_, we observed different degrees of changes in particular high-risk subtypes (**Table 4**).

In clinical practice, when the EBSLN crosses the STV less than 1 cm above the upper edge of the thyroid superior pole (Cernea 2A and 2B), the nerve is at higher risk of injury than other subtypes of Cernea classification. When the nerve runs into the pharyngeal muscle surface (Friedman 1), it is not an indicator of a high-risk condition. However, Friedman subtype 2 is more likely to have a higher risk of EBSLN injury (**Table 3**). Compared to other subtypes, Kierner 4 is a rare type with a high risk of injury.

Moreover, this is the first research that has analyzed which EBSLN classification is the most useful for predicting high-risk conditions. We found the risk prediction of the Friedman scheme was greater than Cernea and Kierner. Surgeons should pay more attention to the relationship between the EBSLN and STV. The STV should not be blindly ligated before confirming the relationship with the nerve.

Furthermore, the assessment of EBSLN by CTM twitch was higher than the EMG response. In our study, the identification by CTM twitch was 100%, but the EMG response was 91.2%. Related research pointed out that the identification by CTM twitch was 100%, and the EMG response was 74-100% (1, 14–19). The variability may be due to a) the presence of the human communicating nerve (20, 21), b) EMG tube position, c) the limited area of muscle activity induced, and d) intrinsic EBSLN low amplitude and short latency profiles. Therefore, we recommend that the EBSLN is identified by CTM twitch response. EMG is only for reference and not the standard for identifying EBSLN.

During the study, we faced two LOS of EBSLN, in S2 determination. The EBSLN guidelines (1) were published in 2013, but the LOS of EBSLN has not been defined, due to the EBSLN amplitude being lower and individual variation being larger than RLN. In our study, the difference among the Cernea subtypes was found to be significant by comparing the amplitude of EBSLNs and vocal acoustic assessment (**Table 3**).

This might result in the definition of EBSLN LOS. Unlike RLN, the amplitude of EBSLN is lower and with larger individual variation. In this study, the EBSLN amplitude was 225 ± 162μV, Dionigi et al. (13) concluded from 400 EBSLNs that the mean amplitude was 259 ± 67 (180-421), 321 ± 79 (192-391), 371 ± 38 (200-551)μV, respectively, for type 1, 2A, 2B of Cernea classification. Barczynski et al. (14) found the mean EBSLN amplitude was equal to 249 ± 144μV. Randolph et al. (22) concluded from 73 EBSLN studies that the EBSLN amplitude was 269 ± 176μV. Thus, the cut-off value of EBSLN may be lower than that of RLN (50%).

Finally, EBSLN damage rates are different with distinct diagnostic criteria, from 0.45% to 58% (23–29) (**Table 6**). In this study, high-risk subtypes showed significant differences in the maximum frequency, range of frequency, and maximum frequency duration, which may indicate the existence of dysfunction. However, the postoperative symptoms of EBSLN injury are similar to symptoms of laryngeal and vocal cord edema caused by intubation technology and too long surgery time. It may be useful to evaluate the EBSLN injury rate through both intraoperative EMG parameters combined with vocal acoustic assessment and stroboscopy (30).

**Table 6:** EBSLN injury rates and diagnostic criteria.

Author	The rate of EBSLN injury (%)	Diagnostic criteria
EI-Guindy (24)	2.4%	Vocal acoustic assessment
Hunt (25)	0.45%	Laryngoscope
Dener (26)	0.9%	Not reported
Kark (27)	18%	Voice handicap index
Lennquist (28)	2.0%	Laryngoscope
Jansson (23)	58%	EMG
Aluffi (29)	14%	EMG

A limitation of this study is the absence of incorporating CTM electromyography as a reference for evaluating the incidence of EBSLN injury. Additionally, there was a lack of assessment of other potential risk factors for recurrent laryngeal nerve injury, such as thyroid volume, neck length, and distance of the tumor from the upper pole of the thyroid. These variables could potentially influence the risk of nerve injury and should be considered in future research. Acknowledging these limitations, we aim to address these factors in future studies.

## Data availability statement

The raw data supporting the conclusions of this article will be made available by the authors, without undue reservation.

## Ethics statement

The studies involving humans were approved by the Institutional Review Board of the China-Japan Union Hospital of Jilin University. The studies were conducted in accordance with the local legislation and institutional requirements. Written informed consent for participation was not required from the participants or the participants’ legal guardians/next of kin in accordance with the national legislation and institutional requirements. Patients signed an informed consent before surgery.

## Author contributions

CL: Writing – original draft. JZ: Writing – review & editing. GD: Writing – review & editing. HS: Funding acquisition, Supervision, Writing – review & editing.
